# Neuropsychiatry of Parkinson's Disease

**DOI:** 10.1155/2012/908943

**Published:** 2012-04-17

**Authors:** Antonio L. Teixeira, Leonardo F. Fontenelle, Edward C. Lauterbach, Sergio Starkstein

**Affiliations:** ^1^Neurology Section, Department of Internal Medicine, School of Medicine, Federal University of Minas Gerais, 30130-100 Belo Horizonte, MG, Brazil; ^2^Department of Psychiatry and Legal Medicine, School of Medicine, Federal University of Rio de Janeiro, 22290-140 Rio de Janeiro, RG, Brazil; ^3^Department of Psychiatry and Behavioral Sciences and Department of Internal Medicine (Neurology Section), Mercer University School of Medicine, Macon, GA 31201, USA; ^4^Neuropsychiatry Unit, Fremantle Hospital, School of Psychiatry and Clinical Neurosciences, University of Western Australia, Fremantle, WA 6959, Australia

Parkinson's disease (PD) is traditionally regarded as a motor or a movement disorder. In recent years, however, an increased interest has been directed to its nonmotor symptoms as they seem to determine significant disability at all stages of the disease. Some PD patients consider their nonmotor symptoms even more disabling than motor signs. Among these nonmotor symptoms, neuropsychiatric syndromes (or behavioral and psychological symptoms) are of paramount importance. They include a wide array of syndromes such as anxiety, depression, psychosis, impulse control disorders, apathy, and cognitive dysfunction. Their pathogenesis in PD is rather complex, involving neurodegenerative, drug-related, and psychological mechanisms. Their recognition and management represent a great challenge.

The paper by R. de la Fuente-Fernández presents a staging model named PD-related frontostriatal cognitive dysfunction (PDFCD) that provides a clinical and hypothesis-testing framework for neuropsychiatric syndromes in PD. De La Fuente-Fernandez's PDFCD model proposes three stages based on the sequential process of dopamine depletion occurring in different regions of the striatum (stages I and II) and the frontal cortex (stage III). Therefore, PD patients would present executive dysfunction and mental fatigue (stage I), depression/anxiety (stage IIa), apathy/pain (stage IIb), and dementia (stage III).

The paper by J. Calleo et al. summarizes the current literature on the cognitive rehabilitation programs in PD patients. Despite limited available data and heterogeneity of motor and nonmotor symptoms in PD, cognitive rehabilitation seems to be promising in this context.

The paper by Y. Bogdanova and A. Cronin-Golomb1 presents an original study that assessed cognitive correlates of anxiety and apathy in PD patients. In comparison with matched controls, PD patients exhibited higher levels of anxiety and apathy which were correlated with disease duration. Interestingly, anxiety and apathy correlated differently with cognitive functions. The question remains open whether the treatment of both conditions impacts on cognitive functioning in PD.

Until recently, most studies have focused on the impact of motor disability and depressive symptoms on the quality of life of PD patients, neglecting the role of anxiety. The paper by K. K. Hanna and A. Cronin-Golomb addressed this issue, reporting that anxiety, more than depressive, cognitive, and motor symptoms, significantly affected quality of life in PD.

Dopaminergic strategies remain the mainstay of PD treatment. However, they are frequently associated with neuropsychiatric syndromes, notably psychosis. Alternative therapeutic options have been searched in the last years, and adenosine A_2A_ receptor antagonists seem to be very promising. The paper by C. J. Bleickardt et al. demonstrates that, in comparison with dopamine receptor agonists, adenosine A_2A_ antagonists exhibit better neuropsychiatric profiles as they do not interfere with prepulse inhibition in rodents (a model of psychosis). 

The paper by E. C. Lauterbach et al. presents a comprehensive review of the neuroprotective effects of the psychotropic drugs used in PD.

*Antonio L. Teixeira* *Antonio L. Teixeira* *Leonardo F. Fontenelle* *Leonardo F. Fontenelle* *Edward C. Lauterbach* *Edward C. Lauterbach* *Sergio Starkstein* *Sergio Starkstein*

